# Monocyte-derived extracellular Nampt-dependent biosynthesis of NAD^+^ protects the heart against pressure overload

**DOI:** 10.1038/srep15857

**Published:** 2015-11-02

**Authors:** Masamichi Yano, Hiroshi Akazawa, Toru Oka, Chizuru Yabumoto, Yoko Kudo-Sakamoto, Takehiro Kamo, Yu Shimizu, Hiroki Yagi, Atsuhiko T. Naito, Jong-Kook Lee, Jun-ichi Suzuki, Yasushi Sakata, Issei Komuro

**Affiliations:** 1 Department of Cardiovascular Medicine, Osaka University Graduate School of Medicine, Suita, Osaka 565-0871, Japan; 2 Department of Cardiovascular Medicine, Graduate School of Medicine, The University of Tokyo, Bunkyo-ku, Tokyo 113-8655, Japan; 3 Clinical Pharmacy Education Unit, Graduate School of Pharmaceutical Sciences, Osaka University, Suita, Osaka 565-0871, Japan; 4 Department of Cardiovascular Regenerative Medicine, Osaka University Graduate School of Medicine, Suita, Osaka 565-0871, Japan; 5 Department of Advanced Clinical Science and Therapeutics, Graduate School of Medicine, The University of Tokyo, Bunkkyo-ku, Tokyo 113-8655, Japan; 6 AMED-CREST, Japan Agency for Medical Research and Development, Chiyoda-ku, Tokyo 100-0004, Japan

## Abstract

Nicotinamide phosphoribosyltransferase (Nampt) catalyzes the rate-limiting step in the salvage pathway for nicotinamide adenine dinucleotide (NAD^+^) biosynthesis, and thereby regulates the deacetylase activity of sirtuins. Here we show accommodative regulation of myocardial NAD^+^ by monocyte-derived extracellular Nampt (eNampt), which is essential for hemodynamic compensation to pressure overload. Although intracellular Nampt (iNampt) expression was decreased in pressure-overloaded hearts, myocardial NAD^+^ concentration and Sirt1 activity were preserved. In contrast, iNampt was up-regulated in spleen and monocytes, and circulating eNampt protein and nicotinamide mononucleotide (NMN), a key precursor of NAD^+^, were significantly increased. Pharmacological inhibition of Nampt by FK866 or depletion of monocytes/macrophages by clodronate liposomes disrupted the homeostatic mechanism of myocardial NAD^+^ levels and NAD^+^-dependent Sirt1 activity, leading to susceptibility to cardiomyocyte apoptosis and cardiac decompensation in pressure-overloaded mice. These biochemical and hemodynamic defects were prevented by systemic administration of NMN. Our studies uncover a crucial role of monocyte-derived eNampt in myocardial adaptation to pressure overload, and highlight a potential intervention controlling myocardial NAD^+^ against heart failure.

Nicotinamide adenine dinucleotide (NAD^+^) is an oxidoreductase cofactor for many metabolic reactions such as glycolysis, fatty acid β-oxidation, the TCA cycle, and mitochondrial oxidative phosphorylation^1^. NAD^+^ also functions as an essential substrate for enzymes such as poly(ADP-ribose) polymerases^2^ and sirtuins^3^. Sirtuins are NAD^+^-dependent enzymes that catalyze deacetylation of histones and a wide variety of proteins in multiple cellular compartments^4^, and the mammalian sirtuins (comprising Sirt1-Sirt7) act as pivotal regulators of maintenance of energy homeostasis and prevention of aging-related diseases^4^. Accordingly, tight regulation of intracellular NAD^+^ levels is critical for cellular and organismal survival under pathophysiological conditions. NAD^+^ can be synthesized *de novo*, but the majority of NAD^+^ is synthesized by the salvage pathway from its precursors nicotinic acid, nicotinamide (NAM), or NAM ribose^5^. In mammalian cells, conversion of NAM to NAM mononucleotide (NMN) by NAM phosphoribosyltransferase (Nampt) is the rate-limiting step of this pathway^6^. Intriguingly, Nampt exists not only as an intracellular form (iNampt) but also as an extracellular form (eNampt) in an enzymatically active dimer^7^, and eNampt, previously named pre-B cell colony-enhancing factor or visfatin, is produced and secreted by adipocytes, mononuclear cells, hepatocytes, and cardiomyocytes^7^^-^9. A high concentration of NMN is present in mouse plasma, and eNampt-mediated extracellular production of NMN regulates intracellular NAD^+^ biosynthesis in metabolic tissues such as liver and white adipose tissue in high-fat diet-induced diabetic mice^10^.

In the heart, the expression level of Nampt protein was down-regulated under pathological conditions such as ischemia, ischemia/reperfusion, and pressure overload^11^. However, controversy exists over whether Nampt is beneficial or detrimental to cardiac pathophysiology^9^,^11^,^12^, and it remains unclear how myocardial NAD^+^ biosynthesis is regulated by iNampt and eNampt under pathological conditions. Here we demonstrate that myocardial NAD^+^ concentration is preserved in spite of decreased iNampt expression in mice after transverse aortic constriction (TAC). Mechanistically, monocyte-derived eNampt contributes to preservation of myocardial NAD^+^ levels that is sufficient for the functional compensation to pressure overload. Our studies provide mechanistic insights into the inter-tissue regulation of cardiac homeostasis involving bone marrow-derived monocytes, and point toward a therapeutic strategy of manipulating this pathway for treatment of heart failure.

## Results

### Nampt-dependent biosynthesis of NAD^+^ protects rat neonatal cardiomyocytes from doxorubicin-induced cardiotoxicity

**Figure 1:**
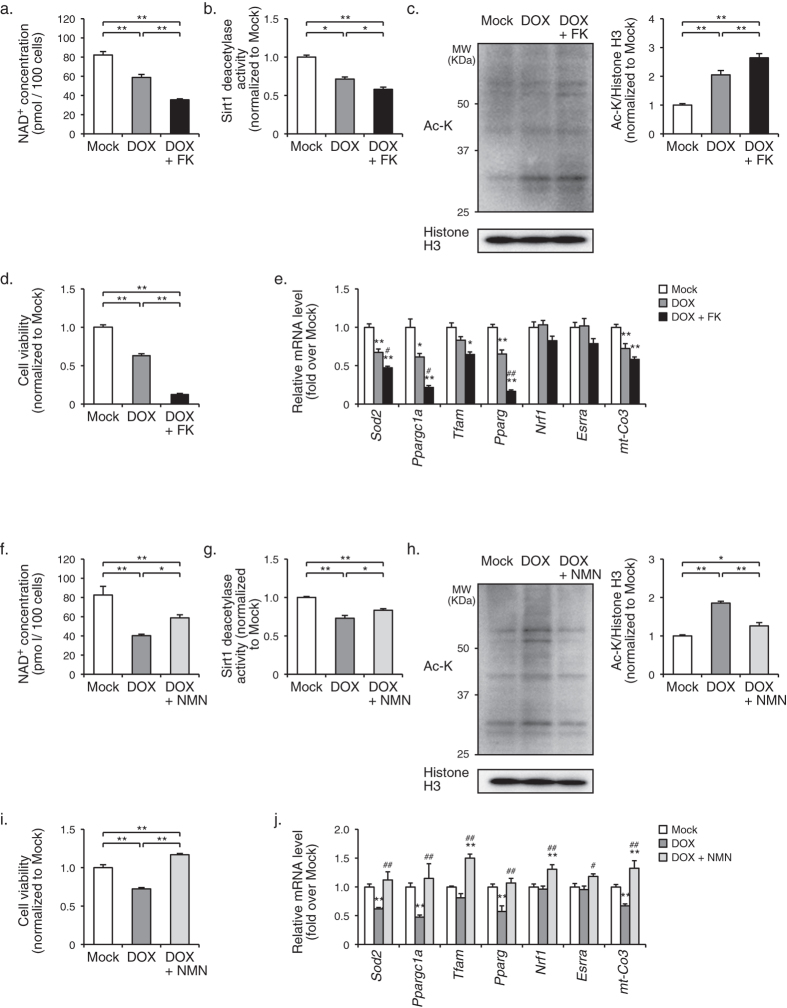
Protective role of Nampt-dependent biosynthesis of NAD^+^ and Sirt1 activation against DOX-induced toxicity in rat neonatal cardiomyocytes. (**a**) NAD^+^ concentrations in cardiomyocytes treated with mock (*n* = 7), DOX (*n* = 7), or DOX + FK866 (*n* = 11). **P < 0.01. (**b**) Sirt1 deacetylase activity in cardiomyocytes treated with mock (*n* = 4), DOX (*n* = 5), or DOX + FK866 (*n* = 5). *P < 0.05, **P < 0.01. (**c**) Immunoblot analysis of acetylated lysine (Ac-K) and histone H3 in the nuclear fraction of cardiomyocytes treated with mock, DOX, or DOX + FK866. The quantitations of Ac-K/histone H3 are shown as bar graphs (*n* = 8, in each group). **P < 0.01. (**d**) Cell viability in cardiomyocytes treated with mock, DOX, or DOX + FK866 (*n* = 6, in each group). **P < 0.01. (**e**) The mRNA levels of mitochondria-associated genes in cardiomyocytes treated with mock (*n* = 7), DOX (*n* = 7), or DOX + FK866 (*n* = 5). *P < 0.05 versus mock, **P < 0.01 versus mock, ^#^*P* < 0.05 versus DOX, ^##^*P* < 0.01 versus DOX. (**f**) NAD^+^ concentrations in cardiomyocytes treated with mock (*n* = 4), DOX (*n* = 3), or DOX + NMN (*n* = 4). *P < 0.05, **P < 0.01. (**g**) Sirt1 deacetylase activity in cardiomyocytes treated with mock (*n* = 4), DOX (*n* = 5), or DOX + NMN (*n* = 5). *P < 0.05, **P < 0.01. (**h**) Immunoblot analysis of Ac-K and histone H3 in cardiomyocytes treated with mock, DOX, or DOX + NMN. The quantitations of Ac-K/histone H3 are shown as bar graphs (*n* = 8, in each group). *P < 0.05, **P < 0.01. (**i**) Cell viability in cardiomyocytes treated with mock, DOX, or DOX + NMN (*n* = 6, in each group). **P < 0.01. (**j**) The mRNA levels of mitochondria-associated genes in cardiomyocytes treated with mock (*n* = 7), DOX (*n* = 5), or DOX + NMN (*n* = 5). **P < 0.01 versus mock, ^#^*P* < 0.05 versus DOX, ^##^*P* < 0.01 versus DOX.

We first examined the impact of Nampt-dependent NAD^+^ biosynthesis on stress response to injury in cultured cardiomyocytes of neonatal rats. Doxorubicin (DOX), an anthracycline anticancer drug, has cardiotoxicity through multiple mechanisms including an increase in oxidative stress, myofibrillar deterioration, and intracellular Ca^2+^ dysregulation^13^. Stimulation with DOX significantly decreased NAD^+^ concentration and enzymatic activity of Sirt1, and conversely increased acetylation of nuclear proteins in cardiomyocytes, which was further exaggerated by concomitant treatment with FK866, a selective Nampt inhibitor^14^ (Fig. 1a–c). It has been reported that Sirt1 regulates mitochondrial function and energy utilization, and protects cardiomyocytes from cell death and degeneration^15^,^16^. Consistently, DOX-induced decrease in cell viability and in expression levels of genes relevant to mitochondrial function, such as *Sod2*, *Ppargc1a*, *Pparg*, and *mt-Co3*, were further exaggerated by treatment with FK866 (Fig. 1d,e). Conversely, treatment of DOX-stimulated cardiomyocytes with NMN significantly increased NAD^+^ concentration, enzymatic activity of Sirt1, decreased acetylation of nuclear proteins, and restored cell viability and the expression levels of mitochondrial genes (Fig. 1f–j). These results suggest that Nampt-dependent biosynthesis of NAD^+^ is protective against DOX-induced cardiotoxicity in cultured cells.

### Myocardial NAD^+^ concentration and Sirt1 activity are preserved in spite of decreased iNampt expression in TAC-operated mouse hearts

**Figure 2:**
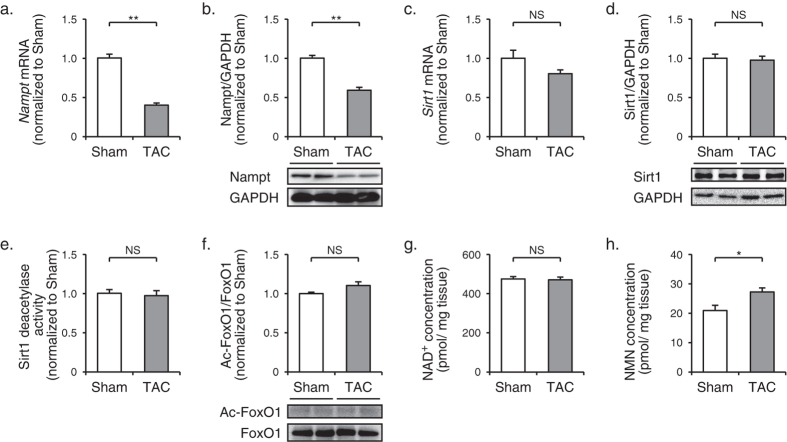
Compensated myocardial NAD^+^ concentration and Sirt1 activity in spite of decreased Nampt expression in TAC-operated hearts. (**a**) The mRNA levels of *Nampt* in hearts at 8 w after TAC or sham operation (*n* = 5). (**b**) Immunoblot analysis of Nampt in hearts at 8 w after operation. The quantitations of the Nampt/GAPDH are shown as bar graphs (*n* = 5). (**c**) The mRNA levels of *Sirt1* in hearts at 8 w after operation (*n* = 5). (**d**) Immunoblot analysis of Sirt1 in hearts at 8 w after operation. The quantitations of the Sirt1/GAPDH are shown as bar graphs (*n* = 5). (**e**) Sirt1 deacetylase activity in hearts at 8 w after operation (TAC, *n* = 5; sham; *n* = 8). (**f**) Immunoblot analysis of acetylated FoxO1 (Ac-FoxO1) and FoxO1 in hearts at 8 w after operation. The quantitations of the Ac-FoxO1/FoxO1 are shown as bar graphs (*n* = 4). (**g**) NAD^+^ concentrations (TAC, *n* = 10; sham, *n* = 11) and (H) NMN concentrations (TAC, *n* = 5; sham, *n* = 4) in hearts at 8 w after operation. *P < 0.05, **P < 0.01, NS, not significant.

Next, to examine the pathophysiological connection between Nampt-dependent NAD^+^ biosynthesis and heart failure, we imposed pressure overload on mice by producing TAC. In this model, we were able to induce adaptive cardiac hypertrophy with preserved systolic function at 2 w, and maladaptive and heart failure at 8 w after operation (Supplementary Table 1). At 8 w after operation, mRNA and protein levels of Nampt were significantly decreased in TAC-operated hearts compared with sham-operated hearts (Fig. 2a,b). But surprisingly, enzymatic activity as well as mRNA and protein levels of Sirt1, and acetylation level of forkhead box protein O1 (FoxO1) were unchanged between TAC- and sham-operated hearts (Fig. 2c–f). In spite of decrease in iNampt protein levels, NAD^+^ concentration was preserved in TAC-operated hearts, as revealed by direct measurement using an HPLC system (Fig. 2g). We also found that NMN concentration was significantly higher in TAC-operated hearts than that in sham-operated hearts (Fig. 2h). These results suggest that an increase in myocardial supply of NMN might compensate for a decrease in iNampt-dependent synthesis of NMN to achieve the steady concentration of NAD^+^ in TAC-operated hearts.

### Nampt protein expression is increased in spleen and circulating CD11b^+^ monocytes in TAC-operated mice.

**Figure 3:**
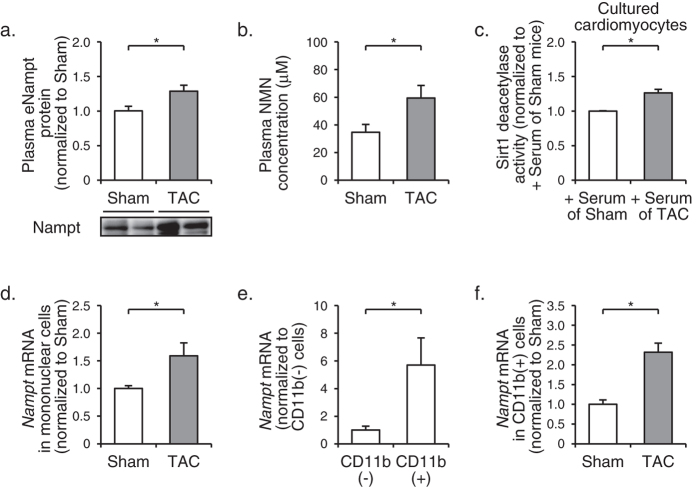
CD11b + monocytes as possible sources of increased eNampt protein in peripheral blood of TAC-operated mice. (**a**) Immunoblot analysis of eNampt in plasma at 8 w after TAC (*n* = 16) or sham (*n* = 17) operation. The quantitations of the eNampt are shown as bar graphs. (**b**) NMN concentrations in plasma at 8 w after operation (*n* = 6). (**c**) Sirt1 deacetylase activity in rat neonatal cardiomyocytes treated with serum of mice at 8 w after TAC (*n* = 10) or sham (*n* = 3) operation. (**d**) The mRNA levels of *Nampt* in mononuclear cells isolated from peripheral blood at 8 w after TAC (*n* = 8) or sham (*n* = 9) operation. (**e**) The mRNA levels of *Nampt* in CD11b – and CD11b + cells in peripheral blood (*n* = 3). Data are shown as fold induction over CD11b – cells. (**f**) The mRNA levels of *Nampt* in CD11b + cells in peripheral blood at 8 w after TAC (*n* = 10) or sham (*n* = 5) operation. *P < 0.05.

In TAC-operated mice, both plasma levels of eNampt protein and NMN were significantly increased, as compared with sham-operated mice (Fig. 3a,b), suggesting that the increase in eNampt-dependent biosynthesis of plasma NMN may contribute to the increase in myocardial NMN levels in TAC-operated mice. In addition, treatment of rat neonatal cardiomyocytes with TAC-operated mouse serum induced significantly higher deacetylase activity of Sirt1 than that with sham-operated mouse serum (Fig. 3c), suggesting that increase in eNampt-dependent biosynthesis of plasma NMN was sufficient for activation of Sirt1 in myocardium.

In an attempt to search for the possible sources of increased plasma eNampt, we examined expression levels of Nampt protein in mouse tissues after TAC or sham operation. Among the tissues examined, only spleen showed a significant increase in Nampt expression after TAC operation (Supplementary Figure 1). We also found that the expression levels of *Nampt* mRNA were significantly increased in mononuclear cells isolated from peripheral blood of TAC-operated mice (Fig. 3d). To determine which subpopulation potentially contributes to the increased *Nampt* expression in peripheral mononuclear cells, we separated cells from peripheral mononuclear cells of unoperated mice using myelomonocytic lineage marker CD11b (Supplementary Figure 2a), and found that *Nampt* mRNA expressions were significantly higher in CD11b + cells than in CD11b - cells (Fig. 3e). Furthermore, *Nampt* mRNA expressions in CD11b + cells from TAC-operated mice were significantly higher than those from sham-operated mice (Fig. 3f). CD11b + cells isolated from peripheral mononuclear cells showed high expression levels of *Emr1* mRNA (Supplementary Figure 2b), and thus were considered to be CD11b+, F4/80+ monocytes. Since spleen is a reservoir for extramedullary monocytes that are mobilized in response to myocardial ischemia^17^, we speculated that monocytes might be the possible source of increased plasma eNampt protein.

### Nampt-dependent biosynthesis of myocardial NAD^+^is essential for functional compensation to pressure overload in mice

**Figure 4:**
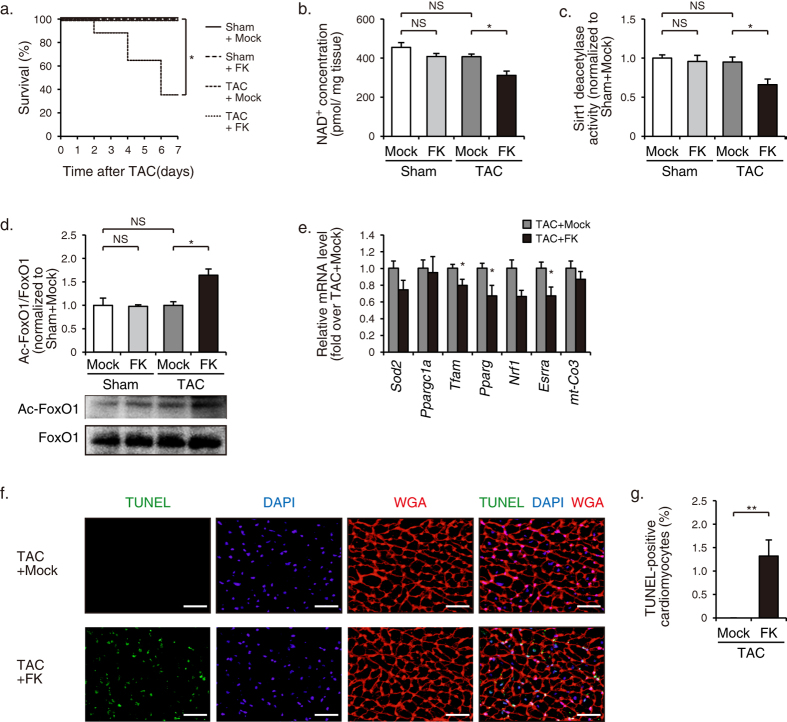
Cardiac decompensation by treatment with FK866 in pressure-overloaded mice. (**a**) Kaplan-Meier survival curves of mice treated with FK866 (FK) or mock after TAC or sham operation (sham + mock, *n* = 5; sham + FK, *n* = 5; TAC + mock, *n* = 8; TAC + FK, *n* = 17). (**b**) NAD^+^ concentrations in hearts at 7 d after operation (sham + mock, *n* = 3; sham + FK, *n* = 3; TAC + mock, *n* = 8; TAC + FK, *n* = 5). (**c**) Sirt1 deacetylase activity in hearts at 7 d after operation (sham + mock, *n* = 4; sham + FK, *n* = 4; TAC + mock, *n* = 7; TAC + FK, *n* = 5). (**d**) Immunoblot analysis of Ac-FoxO1 and FoxO1 in hearts at 7 d after operation (sham + mock, *n* = 3; sham + FK, *n* = 3; TAC + mock, *n* = 7; TAC + FK, *n* = 5). The quantitations of the Ac-FoxO1/FoxO1 are shown as bar graphs. (**e**) The mRNA levels of mitochondria-associated genes in hearts at 7 d after operation (TAC + mock, *n* = 12; TAC + FK, *n* = 6). (**f**) TUNEL staining (*green*) with nuclear staining with DAPI (*blue*) and WGA staining (*red*) showing the outlines of cardiomyocytes in mice treated with FK866 or mock at 4 d after TAC operation. Scale bars, 40 μm. (**g**) Quantification of TUNEL-positive cardiomyocytes in hearts at 4 d after operation (TAC + mock, *n* = 6; TAC + FK, *n* = 5). *P < 0.05, **P < 0.01, NS, not significant.

To examine functional significance of Nampt in hemodynamic compensation to pressure overload, we intraperitoneally administered FK866 or mock to TAC- or sham-operated mice. Since a decrease in cardiac iNampt expression and an increase in plasma eNampt expression were observed as early as at 1 w after TAC operation (Supplementary Figure 3), we started administration of FK866 on the day of operation. Echocardiographic examination revealed that administration of FK866 for 1 w induced a significant increase in left ventricular end-diastolic dimension (LVEDD) and a significant decrease in fractional shortening (FS) in TAC-operated mice, while these parameters were unchanged in sham-operated mice (Supplementary Table 2). As a consequence, approximately 65% of TAC-operated mice died within 1 w after treatment with FK866 (Fig. 4a). In TAC-operated mice, FK866 treatment induced a significant decrease in myocardial concentration of NAD^+^ (Fig. 4b) and deacetylase activity of Sirt1 (Fig. 4c), and an increase in acetylation level of FoxO1 (Fig. 4d), although these changes were not observed in sham-operated mice (Fig. 4b–d). As a consequence, FK866-induced reduction of NAD^+^ led to a significant decrease in expression levels of genes relevant to mitochondrial function, such as *Tfam*, *Pparg*, and *Esrra* (Fig. 4e), and a significant increase in the number of TUNEL-positive cardiomyocytes (Fig. 4f,g). These results suggest that functional inhibition of Nampt disrupted the homeostatic mechanism of myocardial NAD^+^ levels and NAD^+^-dependent Sirt1 activity, and thereby induced cardiac decompensation in pressure-overloaded mice.

**Figure 5:**
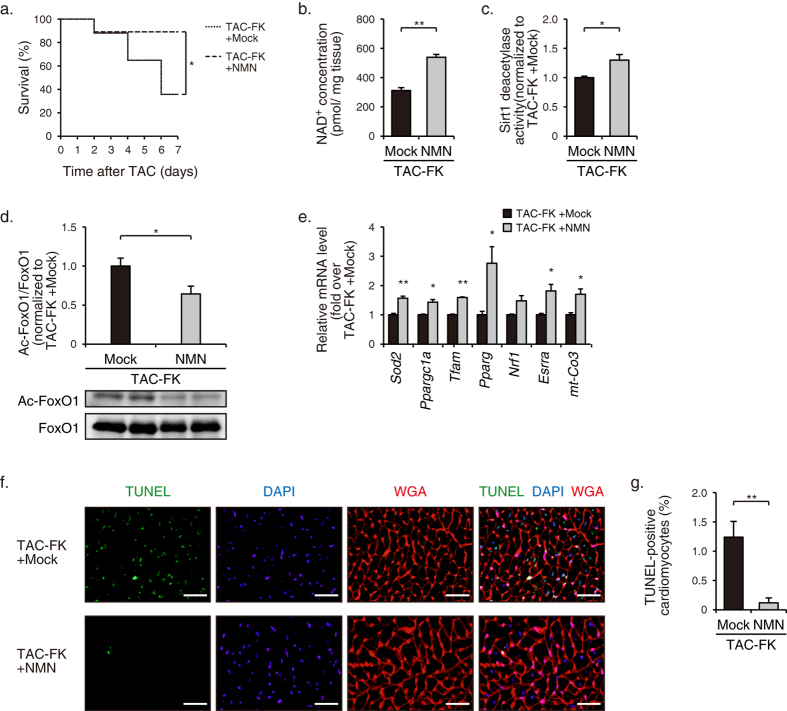
Prevention of FK866-induced cardiac decompensation by NMN administration in pressure-overloaded mice. (**a**) Kaplan-Meier survival curves of TAC-operated mice treated with FK866 (FK) + NMN (*n* = 9) or FK + mock (*n* = 8). (**b**) NAD^+^ concentrations in hearts at 7 d after operation (*n* = 5). (**c**) Sirt1 deacetylase activity in hearts at 7 d after operation (FK + NMN, *n* = 7; FK + mock, *n* = 6). (**d**) Immunoblot analysis of Ac-FoxO1 and FoxO1 in hearts at 7 d after operation (FK + NMN, *n* = 5; FK + mock, *n* = 5). The quantitations of the Ac-FoxO1/FoxO1 are shown as bar graphs. (**e**) The mRNA levels of mitochondria-associated genes in hearts at 7 d after operation (*n* = 4). (**f**) TUNEL staining (*green*) with nuclear staining with DAPI (*blue*) and WGA staining (*red*) showing the outlines of cardiomyocytes in mice treated with FK + NMN or FK + mock at 4 d after TAC operation. Scale bars, 40 μm. (**g**) Quantification of TUNEL-positive cardiomyocytes in hearts at 4 d after operation (*n* = 5). *P < 0.05, **P < 0.01.

To further assess whether the decrease in myocardial NMN and NAD^+^ levels caused cardiac decompensation in FK866-treated TAC-operated mice, we intraperitoneally administered NMN or mock to these mice. NMN treatment significantly improved echocardiographic parameters such as LVEDD and %FS (Supplementary Table 3), and prevented premature death in FK866-treated TAC-operated mice (Fig. 5a). NMN treatment restored myocardial concentration of NAD^+^ (Fig. 5b), deacetylase activity of Sirt1 (Fig. 5c), acetylation level of FoxO1 (Fig. 5d), expression levels of genes relevant to mitochondrial function, such as *Sod2*, *Ppargc1a*, *Tfam*, *Pparg*, *Esrra*, and *mt-Co3* (Fig. 5e), and the number of TUNEL-positive cardiomyocytes (Fig. 5f,g). Collectively, these results suggest that Nampt-dependent biosynthesis of NAD^+^ is essential for myocardial Sirt1 deacetylase activity and functional compensation to pressure overload in mice.

### Monocyte-derived eNampt is crucial for myocardial synthesis of NAD^+^ sufficient for functional compensation to pressure overload in mice

**Figure 6:**
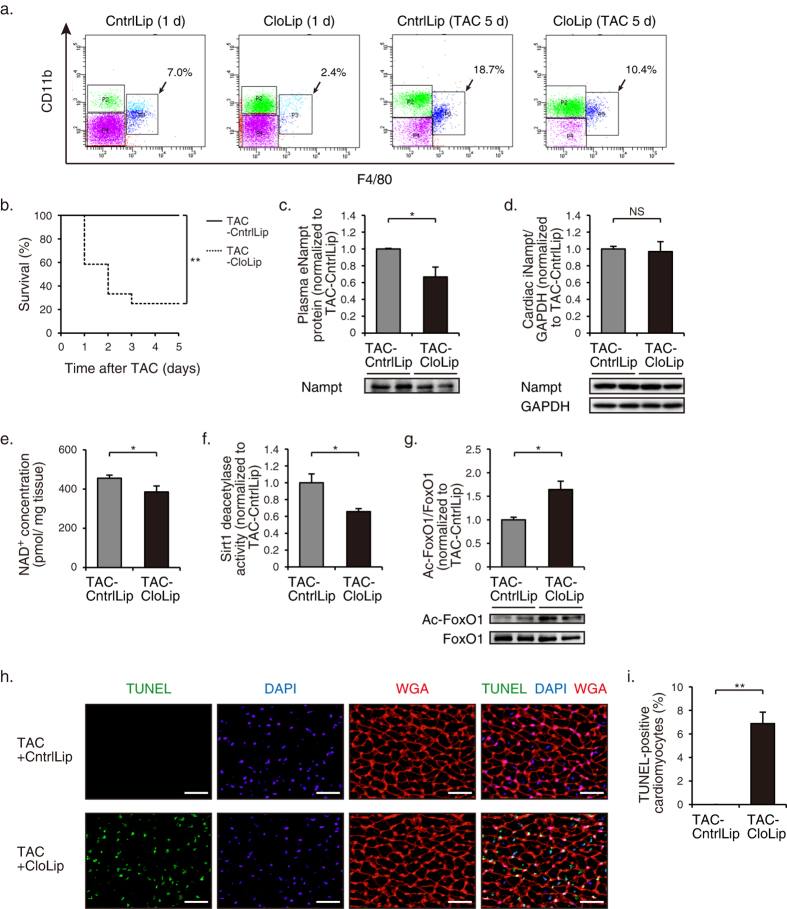
Cardiac decompensation by treatment with CloLip in pressure-overloaded mice. (**a**) Representative flow cytometric analysis demonstrating CD11b+, F4/80 + monocytes in peripheral blood of mice at 1 d after treatment with clodronate liposomes (CloLip) or control liposomes (CntrlLip), and TAC-operated mice at 5 d after operation and treatment with CloLip or CntrlLip. (**b**) Kaplan-Meier survival curves of TAC-operated mice treated with CloLip (*n* = 12) or CntrlLip (*n* = 8). (**c**) Immunoblot analysis of eNampt in plasma at 5 d after TAC operation (CloLip, *n* = 5; CntrlLip, *n* = 4). The quantitations of the eNampt are shown as bar graphs. (**d**) Immunoblot analysis of iNampt in hearts at 5 d after TAC operation (CloLip, *n* = 5; CntrlLip, *n* = 4). The quantitations of the Nampt/GAPDH are shown as bar graphs. (**e**) NAD^+^ concentrations in hearts at 5 d after TAC operation (CloLip, *n* = 5; CntrlLip, *n* = 7). (**f**) Sirt1 deacetylase activity in hearts at 5 d after TAC operation (CloLip, *n* = 5; CntrlLip, *n* = 4). (**g**) Immunoblot analysis of Ac-FoxO1 and FoxO1 in hearts 5 d after TAC operation (CloLip, *n* = 4; CntrlLip, *n* = 4). The quantitations of the Ac-FoxO1/FoxO1 are shown as bar graphs. (**h**) TUNEL staining (*green*) with nuclear staining with DAPI (*blue*) and WGA staining (*red*) showing the outlines of cardiomyocytes in TAC-operated mice at 2 d after operation and treatment with clodronate liposomes (CloLip) or control liposomes (CntrlLip). Scale bars, 40 μm. (**i**) Quantification of TUNEL-positive cardiomyocytes at 2 d after TAC operation (CloLip, *n* = 5; CntrlLip, *n* = 6). *P < 0.05, **P < 0.01, NS, not significant.

The increase in Nampt expression in monocytes and plasma eNampt concentration suggested a causal link between monocyte-derived eNampt and Nampt-dependent functional compensation to pressure overload. To test the importance of monocyte-derived eNampt in this process, we depleted monocytes/macrophages by treatment with clodronate liposomes (CloLip) in mice^18^. We confirmed by flow cytometric analysis that, in unoperated mice, CloLip induced approximately 3.2-fold reduction in the percentage of circulating CD11b+, F4/80 + monocytes at 1 d after the initiation of treatment (Fig. 6a, Supplementary Figure 4a). The percentage of CD11b+, F4/80 + monocytes was increased approximately 3.1-fold at 5 d after TAC operation, but it was decreased approximately 1.6-fold by concomitant CloLip treatment (Fig. 6a, Supplementary Figure 4b). In contrast, the populations of CD11b+, F4/80—cells and CD11b -, F4/80—cells were not significantly changed after CloLip treatment (Supplementary Figure 4). CloLip-treated mice became lethargic and approximately 75% of CloLip-treated mice died within 5 d after TAC operation, although control liposomes (CntrlLip)-treated mice appeared normal (Fig. 6b). Echocardiographic examination revealed a significant increase in LVEDD and a significant decrease in %FS in CloLip-treated mice, while these parameters were unchanged in CntrlLip-treated mice (Supplementary Table 4). CloLip treatment induced a significant decrease in plasma eNampt protein (Fig. 6c) without a significant change in cardiac iNampt protein (Fig. 6d), leading to a significant decrease in NAD^+^ concentration (Fig. 6e) and deacetylase activity of Sirt1 (Fig. 6f), and an increase in acetylation level of FoxO1 (Fig. 6g). CloLip-induced reduction of NAD^+^ led to a significant increase in the number of TUNEL-positive cardiomyocytes (Fig. 6h,i). These results suggest that clodronate liposome-mediated reduction of CD11b + cells decreased plasma eNampt protein, and thereby induced cardiac decompensation in pressure-overloaded mice.

**Figure 7:**
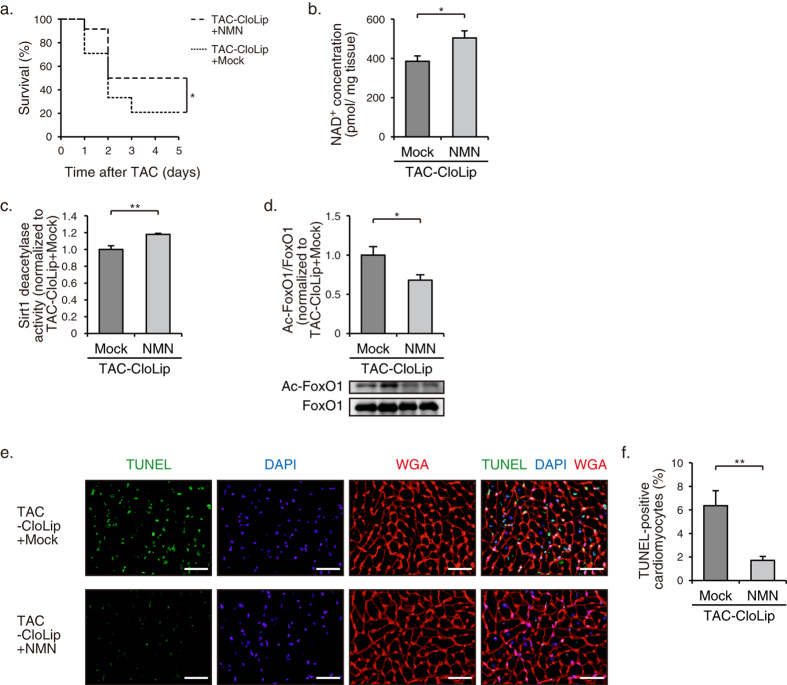
Prevention of CloLip-induced cardiac decompensation by NMN administration in pressure-overloaded mice. (**a**) Kaplan-Meier survival curves of TAC-operated mice treated with CloLip + NMN (*n* = 12) or CloLip + Mock (*n* = 24). (**b**) NAD^+^ concentrations in hearts at 5 d after TAC operation (CloLip + NMN, *n* = 3; CloLip + Mock, *n* = 5). (**c**) Sirt1 deacetylase activity in hearts at 5 d after TAC operation (*n* = 4). (**d**) Immunoblot analysis of Ac-FoxO1 and FoxO1 in hearts at 5 d after TAC operation (*n* = 4). The quantitations of the Ac-FoxO1/FoxO1 are shown as bar graphs. (**e**) TUNEL staining (*green*) with nuclear staining with DAPI (*blue*) and WGA staining (*red*) showing the outlines of cardiomyocytes in TAC-operated mice at 2 d after operation and treatment with CloLip + NMN or CloLip + mock. Scale bars, 40 μm. (**f**) Quantification of TUNEL-positive cardiomyocytes in the hearts at 5 d after TAC operation (*n* = 5). *P < 0.05, **P < 0.01.

We further assessed whether NMN treatment prevented CloLip-induced cardiac decompensation in pressure-overloaded mice. NMN treatment significantly improved echocardiographic parameters such as LVEDD and %FS (Supplementaty Table 4), and prevented premature death in CloLip-treated TAC-operated mice (Fig. 7a). NMN treatment restored myocardial concentration of NAD^+^ (Fig. 7b), deacetylase activity of Sirt1 (Fig. 7c), acetylation level of FoxO1 (Fig. 7d), and the number of TUNEL-positive cardiomyocytes (Fig. 7e,f). Collectively, these results suggest that monocyte-derived eNampt is crucial for myocardial synthesis of NAD^+^ sufficient for functional compensation to pressure overload in mice.

## Discussion

**Figure 8:**
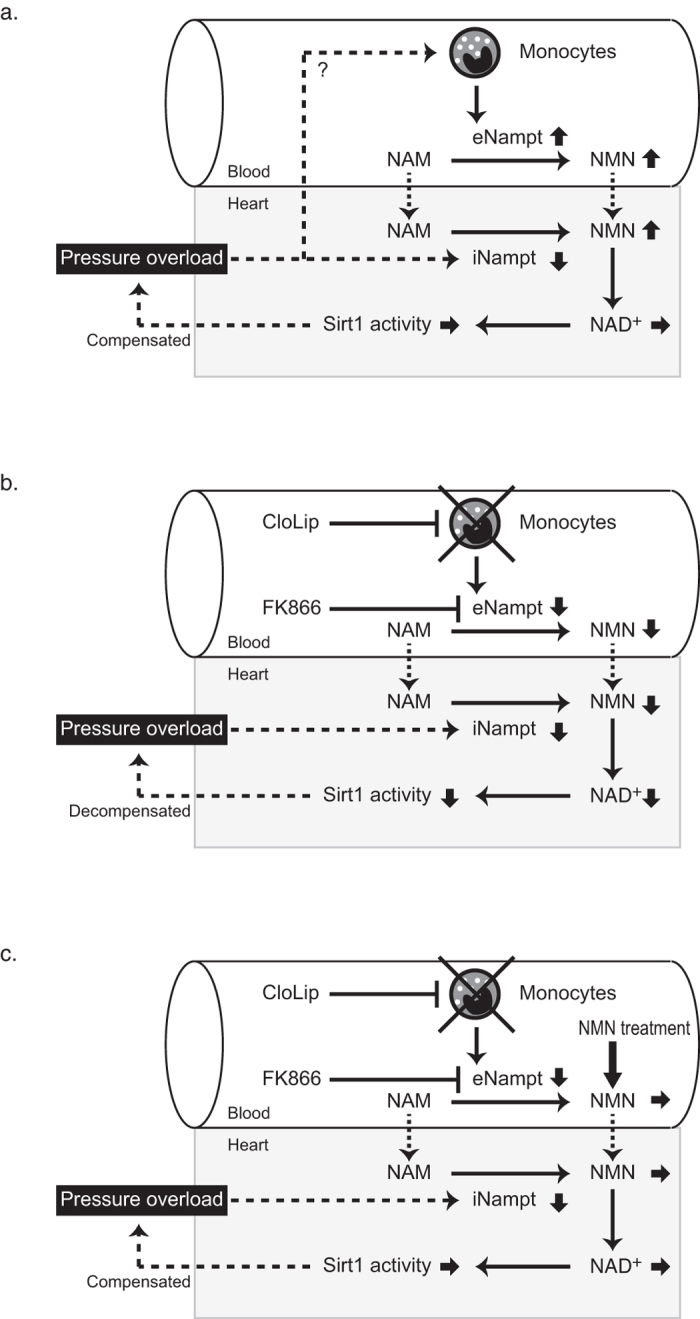
Homeostatic mechanism of monocyte-derived eNampt-dependent biosynthesis of myocardial NAD^+^ in cardiac compensation to pressure overload. (**a**) Pressure overload decreases cardiac iNampt expression, but myocardial NAD^+^ concentration and Sirt1 deacetylase activity are unchanged. Up-regulation of monocyte-derived eNampt contributes to preservation of myocardial NAD^+^ levels and functional compensation to pressure overload. (**b**) Pharmacological inhibition of Nampt by FK866 or depletion of monocytes by CloLip suppresses compensatory up-regulation of monocyte-derived eNampt and disrupts the homeostatic mechanism of myocardial NAD^+^ levels and Sirt1 activity, leading to pressure overload-induced cardiac decompensation. (**c**) Systemic administration of NMN restores myocardial NAD^+^ levels and Sirt1 activity and prevents FK866- or CloLip-induced cardiac decompensation to pressure overload.

Myocardial compensation to increased workload is achieved through complex and integrative processes involving not only a number of intracellular signaling pathways but also cross-talking networks of intercellular and inter-tissue communications. Our present study demonstrates a hitherto unknown mechanism of inter-tissue regulation of cardiac homeostasis by circulating monocytes. We provide proof that an increase in circulating NMN mediated by up-regulation of monocyte-derived eNampt compensates for a decrease in myocardial iNampt expression to maintain the myocardial NAD^+^ level in pressure-overloaded mice (Fig. 8).

It has been reported that down-regulation of Nampt increased apoptotic cell death in cultured neonatal rat cardiomyocytes^11^, and we also observed that Nampt-dependent biosynthesis of NAD^+^ was protective against DOX-induced cardiotoxicity. Cardiac-specific overexpression of Nampt or administration of NMN increased myocardial NAD^+^ levels, and protected the heart from ischemia or ischemia/reperfusion injury in mice, indicating that Nampt is crucial for myocardial NAD^+^ synthesis and cardioprotection in stressed hearts^11^,^12^. On the other hand, Pillai *et al.* reported that cardiac-specific overexpression of Nampt induced cardiac hypertrophy and interstitial fibrosis spontaneously in mice, and that heterozygous knockout of Nampt attenuated isoproterenol- and angiotensin II-induced cardiac hypertrophy, indicating Nampt is a positive regulator of adverse cardiac remodeling^9^. Considering the much greater expression levels of myocardial Nampt in mice reported by Pillai *et al.*, excessive production of Nampt might be detrimental to cardiac pathophysiology. Our present data clearly advance our understanding of hitherto controversial role of Nampt in stress response to hemodynamic overload, demonstrating that inhibition of Nampt induced cardiac decompensation in the early phase of pressure overload.

Recently, eNampt, like cytokines and growth factors, has been reported to act directly on cells, but the actions of eNampt on cardiomyocytes are enigmatic. Treatment with eNampt attenuated H_2_O_2_-induced apoptosis in H9c2 cardiomyocytes^19^, and intravenous administration of eNampt at the time of reperfusion reduced myocardial infarct size in a mouse model of ischemia/reperfusion^20^. On the contrary, Montecucco *et al.* reported that pharmacological inhibition of Nampt reduced neutrophil-mediated myocardial injury in the early phase of reperfusion^21^. It has remained unclear whether monocyte-derived eNampt has biological effects on cardiomyocytes, but we found that plasma NMN was significantly increased in TAC-operated mice, and that treatment of rat neonatal cardiomyocytes with TAC-operated mouse serum induced significantly higher deacetylase activity of Sirt1. Therefore, NMN might be extracellularly converted from NAM by eNampt at a systemic level, and used as a substrate for NAD^+^ biosynthesis after transport flux into the cells via an unidentified NMN transporter in the hearts. Further studies using elaborate genetic models to delete *Nampt* specifically in cardiomyocytes or monocytes will be required to dissect the cooperative regulation of myocardial NAD^+^ by iNampt and eNampt in the hearts subjected to pressure overload and ischemia/reperfusion.

Nampt-dependent NAD^+^ biosynthesis is a key determinant of the deacetylase activity of sirtuins^6^. Our analysis of Sirt1 deacetylase activity revealed that it was invariably correlated with myocardial NAD^+^ concentration both *in vitro* and *in vivo*. In a mouse model of ischemia/reperfusion, cardiac-specific overexpression of Sirt1 reduced infarct size through activation of FoxO1 and manganese superoxide dismutase, while cardiac-specific knockout of Sirt1 exacerbated myocardial injury^22^. However, contradictory phenotypes attributable to gene dosages of the transgene were observed in the development of cardiac hypertrophy in mice with cardiac overexpression of Sirt1. Low to moderate levels of Sirt1 overexpression attenuated aging-dependent progression of cardiac remodeling and dysfunction, but high levels of Sirt1 overexpression increased oxidative stress and apoptosis, leading to cardiac dysfunction^23^,^24^. Beneficial effects of Sirt1 are mediated by a combination of multiple mechanisms such as deacetylation of peroxisome proliferator-activated receptor-γ coactivator-1α for modulating mitochondrial function^16^,^25^, up-regulation of manganese superoxide dismutase for scavenging ROS^22^,^26^, deacetylation of p53 for inhibiting apoptotic cell death^27^, and deacetylation of FoxO1 for inducing autophagy^28^. Conversely, Oka *et al.* reported that overexpressed Sirt1, by forming a complex with peroxisome proliferator-activated receptor-α, suppressed the estrogen-related receptors pathways, leading to mitochondrial dysfunction and progression of heart failure^29^. In TAC-operated mice, the expression levels of genes relevant to mitochondrial function were correlated with Sirt1 deacetylase activity, either when it was decreased by treatment with FK866 or when it was restored by treatment with NMN, supporting the possibility that Nampt-dependent biosynthesis of myocardial NAD^+^ is implicated in the regulation of mitochondrial function. Insomuch as Sirt3 and Sirt7 as well as Sirt1 have been reported to have beneficial roles in the heart^15^, further studies will be required to understand the whole picture of compensatory mechanisms facilitated by eNampt-dependent biosynthesis of NAD^+^ and NAD^+^-dependent activation of Sirt1 and other sirtuins in pressure-overloaded hearts.

In brain and pancreas, basal expression levels of iNampt are extremely low, and circulating NMN maintained by plasma eNampt functions as an essential substrate for intracellular NAD^+^ biosynthesis^7^. Recent studies have demonstrated the effectiveness of NMN supplementation for restoration of NAD^+^ in these tissues^7^,^10^,^30^,^31^. Under the pathological conditions in which circulating NMN levels decline, NMN supplementation may provide therapeutic benefit. It has been reported that the levels of Nampt protein as well as NAD^+^ decline with aging in multiple tissues^10^,^32^. We observed that aged mice showed a significant decrease in the levels of myocardial NAD^+^ and plasma eNampt protein, as compared with young mice, although the levels of myocardial iNampt protein were unchanged (Supplemental Figure S5). It is well known that the prevalence of heart failure increases with age, and that the elderly patients with heart failure have a worse prognosis than the younger ones^33^. It makes sense to hypothesize that age-dependent decline in eNampt-mediated supply of NMN might cause a decrease in myocardial NAD^+^, leading to increased susceptibility to heart failure with higher morbidity and mortality in the elderly. In addition, a recent study demonstrated that high levels of plasma eNampt are associated with favorable clinical outcome in patients with dilated cardiomyopathy^34^, and our study may provide a likely mechanistic explanation for the correlation between plasma eNampt and heart failure phenotypes. Our study highlighted a novel mechanism of circulating eNampt-mediated regulation of myocardial NAD^+^ under stressed conditions, and opened up an opportunity to explore whether restoration of myocardial NAD^+^ has a therapeutic potential for heart failure.

## Methods

### Mice, operation, monocyte/macrophage depletion, and transthoracic echocardiography

All of the experiments were approved by the Institutional Animal Care and Use Committee of Osaka University, and carried out in accordance with the guidelines of Osaka University. C57BL/6J mice were purchased from CLEA Japan, Inc. For TAC operation, we anesthetized 8 to 9-week-old male mice by intraperitoneal injection of medetomidine hydrochloride (0.3 mg/kg), midazolam (4 mg/kg), and butorphanol (5 mg/kg)^35^, and anesthesia was monitored by pinching the toe. Respiration was artificially controlled with a tidal volume of 0.2 ml and a respiratory rate of 110 breaths/min. After median sternotomy, transverse aorta between the branches of right brachiocephalic and left common carotid arteries was constricted with 7–0 silk strings by ligating the aorta with splinting a blunted 27 gauge needle, which was removed after the ligation. The chest was then closed, and mice were allowed to recover from anesthesia while their body temperature was kept at 37 °C. Post-operative analgesia (meloxicam, 5 mg/kg/24 h) was administered subcutaneously for 48 h. The surgeon had no information about the mice used in this study. To inhibit Nampt-dependent biosynthesis of NAD^+^ *in vivo*, we administered FK866 (10 mg/kg/d) or mock (corn oil) by intraperitoneal injection to mice for the duration of the experiment^36^. For NMN treatment, we administered NMN (500 mg/kg) or mock (normal saline) by intraperitoneal injection twice a day for the duration of the experiment^10^,^30^. Clodronate and control liposomes (Clophosome, Combo Kit) were purchased from FormuMax Scientific, Inc. To induce depletion of monocytes and macrophages, a dose of 25 μl of clodronate or control liposomes was administered intraperitoneally. For evaluation of cardiac dimensions and contractility, transthoracic echocardiography was performed on conscious mice with a Vevo 770 Imaging System using a 25 MHz linear probe (Visual Sonics Inc.). Mice were sacrificed to collect tissue samples after 24 h starvation.

### Cell culture and cell viability assay

The experiments for primary cultures of cardiomyocytes was approved by the Animal Study Committee of Osaka University, and were carried out in accordance with the guidelines of Osaka University. Briefly, cervical dislocation euthanasia was performed by a trained personal prior to harvesting the cardiac tissue of 1-day-old Wistar rats according to the American Veterinary Medical Association guidelines for the euthanasia of animals. Ventricles were minced and digested in Ca^2+^-free Hank’s balanced salt solution (HBSS) (Hyclone) containing 0.1% trypsin (Hyclone) and 70 u/ml collagenase (Worthington Biochemical Corp.). Cardiomyocytes were enriched after pre-plating, plated at a field density of 1 × 10^5^ cells/cm^2^, and cultured in DMEM supplemented with 10% fetal bovine serum (BGS) for 24 h. After serum-starvation (1% BGS) for 24 h, cardiomyocytes were stimulated with DOX (1 μM for 3 h) and allosteric Nampt inhibitor FK866 (500 nM for 24 h) or NMN (500 μM for 24 h)^10^. DOX was purchased from Sigma-Aldrich, and FK866 and NMN were purchased from Cayman Chemical and Sigma-Aldrich, respectively. Cell viability was determined using CellTiter 96 AQueous One Solution Cell Proliferation Assay (Promega) that is based on metabolic conversion of a tetrazolium compound MTS to a colored product by living cells.

### Western blot analysis

Cells were lysed and tissues were homogenized in buffer (20 mM Tris, pH 7.4, 150 mM NaCl, 5 mM EDTA, pH 8.0, 100 mM Na_3_VO_4_, 0.5% Nonidet P-40, and Complete Mini protease inhibitor (Roche Applied Science)). For detection of Ac-FoxO1, tissues were homogenized in buffer containing 10 mM NAM and 1 μM trichostatin A. For nuclear fractionation, cells were lysed in buffer (10 mM HEPES, pH 7.9, 1.5 mM MgCl_2_, 10 mM KCl, 0.5 mM DTT, 0.05% Nonidet P-40, 10 mM NAM, 1 μM trichostatin A and Complete Mini protease inhibitor), incubated at 4 °C for 10 min, and centrifuged at 4 °C at 800 *g* for 10 min. Pellet was resuspended in buffer (50 mM Tris, pH8.0, 150 mM NaCl, 0.5% sodium deoxycholate, 1% Nonidet P-40, and 0.1% SDS, 10 mM NAM, 1 μM trichostatin A and Complete Mini protease inhibitor), incubated at 4 °C for 30 min, and centrifuged at 4 °C at 10.400 *g* for 20 min. Supernatant was aliquoted as the nuclear fraction. For preparation of protein samples from mouse plasma, superfluous plasma protein (albumin, IgM, IgG, IgA, haptoglobin, transferrin, α1-antitrypsin, fibrinogen, α 2-macroglobulin, α1-acidglycoprotein, apolipoprotein AI, apolipoprotein AII, complement C3 and transthyretin) was depleted using the Multiple Affinity Removal Spin Cartridge System (Agilent Technologies) according to manufacturer’s instructions.

Protein samples were fractionated with SDS-PAGE, transferred to PVDF membranes (GE Healthcare Biosciences). The blotted membranes were incubated with primary antibody, followed by horseradish peroxidase-conjugated anti-mouse or anti-rabbit IgG antibody (Jackson ImmunoResearch Laboratories, Inc.). Immunoreactive signals were detected with ECL Plus Western Blotting Detection System (GE Healthcare Biosciences), and visualized using a lumino-image analyzer (ImageQuant LAS 4000 mini; GE Healthcare Biosciences). Following primary antibodies were used: rabbit polyclonal anti-PBEF antibody (Bethyl Laboratories, Inc.), rabbit polyclonal anti-Sirt1 antibody (Merck Millipore), rabbit polyclonal anti-GAPDH antibody (Abcam), rabbit polyclonal anti-Ac-FoxO1 antibody (Santa Cruz Biotechnology, Inc.), rabbit monoclonal anti-FoxO1 antibody (Cell Signaling Technology, Inc.), rabbit polyclonal anti-Ac-K antibody (Cell Signaling Technology, Inc.), and rabbit monoclonal anti-histone H3 antibody (Cell Signaling Technology, Inc.).

### Real time RT-PCR analysis

Total RNA was extracted by using the TRIzol Reagent (Life Technologies, Inc.), and treated with DNase to remove contaminating genomic DNA using TURBO DNA-free Kit (Life Technologies, Inc.). Single-stranded cDNA was transcribed by using The SuperScript VILO cDNA Synthesis Kit (Life Technologies, Inc) according to the manufacturer’s protocol. We conducted quantitative real-time PCR analysis using Light Cycler TaqMan Master Kit (Roche Applied Science) with the target-specific primers and the matching probes designed by the Universal ProbeLibrary System (Roche Applied Science), according to the manufacturer’s instructions. Amplification conditions were initial denaturation for 10 min at 95 °C followed by 45 cycles of 10 s at 95 °C and 25 s at 60 °C. Individual PCR products were analyzed by melting-point analysis. The expression level of a gene was normalized relative to that of mouse *Gapdh* and rat 28S rRNA by using a comparative Ct method. The primer sequences and Universal Probe numbers were designed with the ProbeFinder software as following: mouse *Nampt*, 5′-cctgttccaggctattctgttc-3′ and 5′-atggtctttcccccaagc-3′, No. 84; mouse *Sirt1*, 5′-cgtggagacatttttaatcaggta-3′ and 5′-gcttcatgatggcaagtgg-3′, No. 104; mouse *Gapdh,* 5′-tgtccgtcgtggatctgac-3′ and 5′-cctgcttcaccaccttcttg-3′, No. 80; mouse *Sod2*, 5′-gacccattgcaaggaacaa- 3′ and 5′-gtagtaagcgtgctcccacac-3′, No. 3; mouse *Ppargc1a*, 5′-gaaagggccaaacagagaga-3′ and 5′-gtaaatcacacggcgctctt-3′, No. 29; mouse *Tfam*, 5′-caaaggatgattcggctcag-3′ and 5′-aagctgaatatatgcctgcttttg-3′, No. 94; mouse *Pparg*, 5′-aagacaacggacaaatcacca-3′ and 5′-gggggtgatatgtttgaacttg-3′, No. 7; mouse *Nrf-1*, 5′-tggagtccaagatgctaatgg-3′ and 5′-gcgaggctggttaccaca-3′, No.100; mouse *Esrra*, 5′-ccttccctgctggacctc-3′ and 5′-cgacaccagagcgttcact-3′, No. 78; mouse *mt-Co3*, 5′-tagcctcgtaccaacacatga-3′ and 5′-agtggtgaaattcctgttgga-3′, No. 66; mouse *Itgam*, 5′-aaggatgctggggaggtc-3′ and 5′- gtcataagtgacagtgctctggat-3′, No. 16; mouse *Emr1*, 5′-cctggacgaatcctgtgaag-3′ and 5′-ggtgggaccacagagagttg-3′, No. 1; rat 28S rRNA, 5′-gctggctaggcagacaacat-3′ and 5′-gacctgacgatgacagaggaa-3′, No. 107; rat *Sod2*, 5′-tggacaaacctgagccctaa-3′ and 5′-gacccaaagtcacgcttgata-3′, No. 67; rat *Ppargc1a*, 5′-aaagggccaagcagagaga-3′ and 5′-gtaaatcacacggcgctctt-3′, No. 29; rat Tfam, 5′-tcggtcagcatataacatttacg-3′ and 5′-caagcctgatttacaagcttca-3′, No. 79; rat *Pparg*, 5′-cccaatggttgctgattaca-3′ and 5′-ggacgcaggctctactttga-3′, No. 125; rat *Nrf1*, 5′-atagtcctgtctggggaaacc-3′ and 5′-tccatgcatgaactccatct-3′, No. 109; rat *Esrra*, 5′-ggtggacccattgccttt-3′ and 5′-caccagggcgttaactgg-3′, No. 78; rat *mt-Co3*, 5′-taaacccaagcccatgacc-3′ and 5′-agccggatgtaagtagaagagc-3′, No. 92.

### Measurement of NAD^+^ and NMN levels

NAD^+^ and NMN levels were determined using an HPLC system (Shiseido Co., Ltd.) with a CAPCELL PAK C18 MGIII S5 column (15 cm × 2.0 mm; Shiseido) and a YMC-Pack Pro C18 RS column (15 cm × 4.6 mm; YMC Co., Ltd.), respectively, as described previously^10^. Frozen tissues or freshly collected plasma of mice, or cultured cardiomyocytes were extracted in 1 M perchloric acid (200 μl/10 mg tissue, 100 μl/10 μl plasma, 500 μl/6 cm dish). The extracts were centrifuged at 15,000 rpm for 10 min at 4 °C, and the resulting supernatants were neutralized in 3 M K_2_CO_3_ on ice for 10 minutes. After clearing extracts, aliquots of 100 μl were mixed with 50 μl of buffer A (50 mM K_2_HPO_4_/KH_2_PO_4_, pH 7.0) and 50 μl of water. For NAD^+^ measurement, the HPLC was run at a flow rate of 200 μl/min with 100% buffer A from 0–5 min, a linear gradient to 95% buffer A/5% buffer B (100% methanol) from 5–6 min, 95% buffer A/5% buffer B from 6–11 min, a linear gradient to 85% buffer A/15% buffer B from 11–13 min, 85% buffer A/15% buffer B from 13–23 min, and a linear gradient to 100% buffer A from 23–24 min. For NMN measurement, the HPLC was run at a flow rate of 700 μl/min with 100% buffer A from 0–5 min, a linear gradient to 60% buffer A/40% buffer B from 5–10 min, 60% buffer A/40% buffer B from 10–32.5 min, a linear gradient to 100% buffer A from 32.5–35 min. NAD^+^ and NMN were usually eluted at 14 min and 25 min, respectively. NAD^+^ and NMN levels were quantitated based on the peak area compared to a standard curve and normalized to the weight of frozen tissues, the volume of plasma, and the number of cultured cardiomyocytes.

### Assay of Sirt1 activity

Sirt 1 deacetylase activity was determined with the SIRT1 Fluorescent Activity Assay/Drug Discovery Kit (Enzo Life Science International) based on Fluor de Lys–SIRT1 substrate peptide. Protein extracts (10 μg) from mouse hearts or rat neonatal cardiomyocytes were incubated with the fluorogenic acetylated peptide substrate. The reaction was carried out at 37 °C for 1 h, and the fluorescent signal was measured at 360 nm excitation and 460 nm emission on a fluorescence plate reader (SH-9000Lab, Hitachi High-Technologies Corporation).

### Isolation of mononuclear cells and CD11b + monocytes

Mouse peripheral blood was collected, and the mononuclear cells were separated using Histopaque 1083 (Sigma-Aldrich). Briefly, 1ml of whole blood was carefully layered onto 5 ml of Histopaque 1083 in a centrifuge tube, and the tube was centrifuged at 400 *g* for 30 min at room temperature. The opaque interface, containing the mononuclear cell band, was collected using a Pasteur pipet. Separation of CD11b + monocytes was achieved by sorting using the MACS system (Miltenyi Biotech). The mononuclear cells were incubated with rat anti-CD11b antibody (Merck Millipore) for 20 min at 4 °C, washed in PBS supplemented with 3% FBS, incubated with anti-rat micro beads for 20 min at 4 °C, and washed in PBS supplemented with 3% FBS. The samples were passed through a MACS MS column (Miltenyi Biotech) set up in a Miltenyi magnet, and CD11b + monocytes were eluted from the column by washing with PBS supplemented with 3% FBS.

### TUNEL assay

TUNEL assay with nuclear staining with DAPI was performed, using *in situ* Apoptosis Detection Kit (Takara Bio Inc.). Hearts were excised and immediately embedded in Tissue-Tek OCT cryo-compound (Sakura Finetek Japan). Fresh frozen sections at 5 μm were fixed with acetone for 30 min at room temperature, and then washed with PBS for 30 min. The sections were incubated with 0.3% H_2_O_2_ in methanol for 30 min at room temperature to block endogenous peroxidase, and then washed three times with PBS for 5 min. After incubation in permeabilization buffer for 5 min on ice, the sections were put in a humidified chamber, and incubated with TdT enzyme including fluorescein isothiocyanate (FITC)-conjugated dUTP for 60 min at 37 °C. To differentiate between apoptosis in myocytes and non-myocytes, tissue sections were also stained with Alexa Fluor 594-conjugated wheat germ agglutinin (WGA) (Life Technologies, Inc.) for 60 min at room temperature, and then washed three times with PBS for 5 min. Finally the sections were mounted with ProLong Gold Antifade Reagent (Life Technologies, Inc.). Images were acquired with a fluorescence microscope (FSX100; Olympus) and Olympus FSX-BSW software (Olympus).

### Flow cytometric analysis

Whole blood obtained cardiac puncture was buffered with EDTA, subjected to red-cell lysis using BD FACS Lysing Solution (BD Biosciences), and washed in PBS supplemented with 3% fetal calf serum. Blood leukocytes were first incubated with rat anti-mouse FcR/III antibody (2.4G2) (BD Biosciences) to minimize non-specific binding of antibody to FcR. They were further incubated with APC-conjugated anti-CD11b antibody (BD Biosciences) and PE-conjugated anti-F4/80 antibody (BD Biosciences) for 10 min on ice, and washed with PBS supplemented with 3% fetal calf serum. The percentages of CD11b + and F4/80 + cells were analyzed by the FACS Canto II flow cytometer (BD Biosciences) using EXPO32 software (Beckman Coulter).

### Statistics

All of the data are presented as mean ± SEM. Two-group comparison was analyzed by unpaired 2-tailed Student’s *t* test, and multiple-group comparison was performed by1-way ANOVA followed by the Tukey-Kramer HSD test for comparison of means. We estimated survival curves by the Kaplan-Meier method, and compared the groups by generalized Wilcoxon test. Values of *P* < 0.05 were considered statistically significant.

## Additional Information

**How to cite this article**: Yano, M. *et al.* Monocyte-derived extracellular Nampt-dependent biosynthesis of NAD^+^ protects the heart against pressure overload. *Sci. Rep.* **5**, 15857; doi: 10.1038/srep15857 (2015).

## Supplementary Material

Supplementary Information
