# Supplementary material for: Monocyte-derived extracellular Nampt-dependent biosynthesis of NAD+ protects the heart against pressure overload
Source: Sci Rep. 2015 Nov 2;5:15857. doi: 10.1038/srep15857 (PMC4629142; doi:10.1038/srep15857)
Supplement: Supplementary Information [file srep15857-s1.pdf]

Supplementary information

Monocyte-derived extracellular Nampt-dependent biosynthesis of NAD^+ protects the heart against pressure overload

Masamichi Yano, Hiroshi Akazawa, Toru Oka, Chizuru Yabumoto, Yoko Kudo-Sakamoto, Takehiro Kamo, Yu Shimizu, Hiroki Yagi, Atsuhiko T. Naito, Jong-Kook Lee, Jun-ichi Suzuki, Yasushi Sakata, Issei Komuro

Supplementary Table 1. Heart Rates and Echocardiographic Parameters of TAC- or Sham-operated Mice

	Sham (2 w)	TAC (2 w)	Sham (8 w)	TAC (8 w)
Number	6	4	6	6
Heart rate (bpm)	614 ± 16.8	638 ± 10.5	634 ± 17.4	626 ± 11.7
LVEDD (mm)	2.54 ± 0.13	2.58 ± 0.10	2.64 ± 0.10	4.60 ± 0.26 $^{**, ##, ††}$
LVESD (mm)	1.22 ± 0.13	1.15 ± 0.07	1.27 ± 0.11	3.73 ± 0.36 $^{**, ##, ††}$
IVSth (mm)	0.74 ± 0.01	0.97 ± 0.05 **	0.78 ± 0.01 ††	0.74 ± 0.01 ††
PWth (mm)	0.82 ± 0.01	1.09 ± 0.03 **	0.79 ± 0.01 ††	0.79 ± 0.02 ††
FS (%)	52.7 ± 3.18	55.5 ± 2.71	52.5 ± 2.54	19.8 ± 3.89 $^{**, ##, ††}$

Values are mean ± SEM. LVEDD, left ventricular end-diastolic dimension; LVESD, left ventricular end-systolic dimension; IVSth, intraventricular septal thickness; PWth, left ventricular posterior wall thickness; FS, fractional shortening. $^{**}P < 0.01$ versus Sham (2 w); $^{##}P < 0.01$ versus Sham (8 w); $^{††}P < 0.01$ versus TAC (2 w).

Supplementary Table 2. Heart Rates and Echocardiographic Parameters of TAC- or Sham-operated Mice with or without Treatment with FK866

	Sham, Mock	Sham, FK866	TAC, Mock	TAC, FK866
Number	5	4	7	5
Heart rate (bpm)	636 ± 15.8	630 ± 10.5	620 ± 9.20	657 ± 14.5
LVEDD (mm)	2.70 ± 0.04	2.67 ± 0.08	2.57 ± 0.08	2.93 ± 0.10 $^{\#, \dagger}$
LVESD (mm)	1.25 ± 0.09	1.22 ± 0.05	1.15 ± 0.06	1.56 ± 0.11 †
IVSth (mm)	0.66 ± 0.02	0.70 ± 0.01	0.95 ± 0.03 $^{**, \#\#}$	0.86 ± 0.06 $^{**, \#}$
PWth (mm)	0.72 ± 0.01	0.68 ± 0.02	0.98 ± 0.04 $^{**, \#\#}$	0.82 ± 0.05 †
FS (%)	53.5 ± 3.19	54.4 ± 1.35	55.3 ± 1.24	46.4 ± 1.87 †

Values are mean ± SEM. LVEDD, left ventricular end-diastolic dimension; LVESD, left ventricular end-systolic dimension; IVSth, intraventricular septal thickness; PWth, left ventricular posterior wall thickness; FS, fractional shortening. $^{**}P < 0.01$ versus Sham, Mock; $^{\#}P < 0.05$, $^{\#\#}P < 0.01$ versus Sham, FK866; $^{\dagger}P < 0.05$ versus TAC, Mock.

Supplementary Table 3. Heart Rates and Echocardiographic Parameters of TAC-operated Mice Treated with Mock, FK866, and FK866 and NMN

	TAC, Mock	TAC, FK866	TAC, FK866+NMN
Number	7	5	5
Heart rate (bpm)	620 ± 9.25	657 ± 14.5	617 ± 7.65
LVEDD (mm)	2.57 ± 0.08	2.93 ± 0.10 *	2.58 ± 0.08 #
LVESD (mm)	1.15 ± 0.06	1.56 ± 0.11 **	1.18 ± 0.06 #
IVSth (mm)	0.95 ± 0.03	0.86 ± 0.06	0.92 ± 0.04
PWth (mm)	0.98 ± 0.04	0.82 ± 0.05	0.85 ± 0.05
FS (%)	55.3 ± 1.24	46.4 ± 1.87 **	54.4 ± 1.42 #

Values are mean ± SEM. LVEDD, left ventricular end-diastolic dimension; LVESD, left ventricular end-systolic dimension; IVSth, intraventricular septal thickness; PWth, left ventricular posterior wall thickness; FS, fractional shortening. *P < 0.05, **P < 0.01 versus TAC, Mock; $^{\#}P$ < 0.05 versus TAC, FK866.

Supplementary Table 4. Heart Rates and Echocardiographic Parameters of TAC-operated Mice Treated with CloLip + NMN or CloLip + Mock

	TAC CntrlLip+Mock	TAC CloLip+Mock	TAC CloLip+NMN
Number	5	5	5
Heart rate (bpm)	535 ± 13.8	526 ± 8.13	516 ± 10.7
LVEDD (mm)	2.34 ± 0.06	2.89 ± 0.12**	2.27 ± 0.08 ##
LVESD (mm)	1.08 ± 0.05	1.85 ± 0.22**	1.06 ± 0.06 ##
IVSth (mm)	0.90 ± 0.05	0.85 ± 0.02	0.91 ± 0.05
PWth (mm)	0.87 ± 0.02	0.85 ± 0.03	0.93 ± 0.05
FS (%)	53.7 ± 1.35	36.6 ± 4.44**	53.1 ± 2.00 ##

Values are mean ± SEM. LVEDD, left ventricular end-diastolic dimension; LVESD, left ventricular end-systolic dimension; IVSth, intraventricular septal thickness; PWth, left ventricular posterior wall thickness; FS, fractional shortening. $^{**}P < 0.01$ versus TAC-CntrlLip+Mock, $^{\#\#}P < 0.01$ versus TAC-CloLip+Mock.

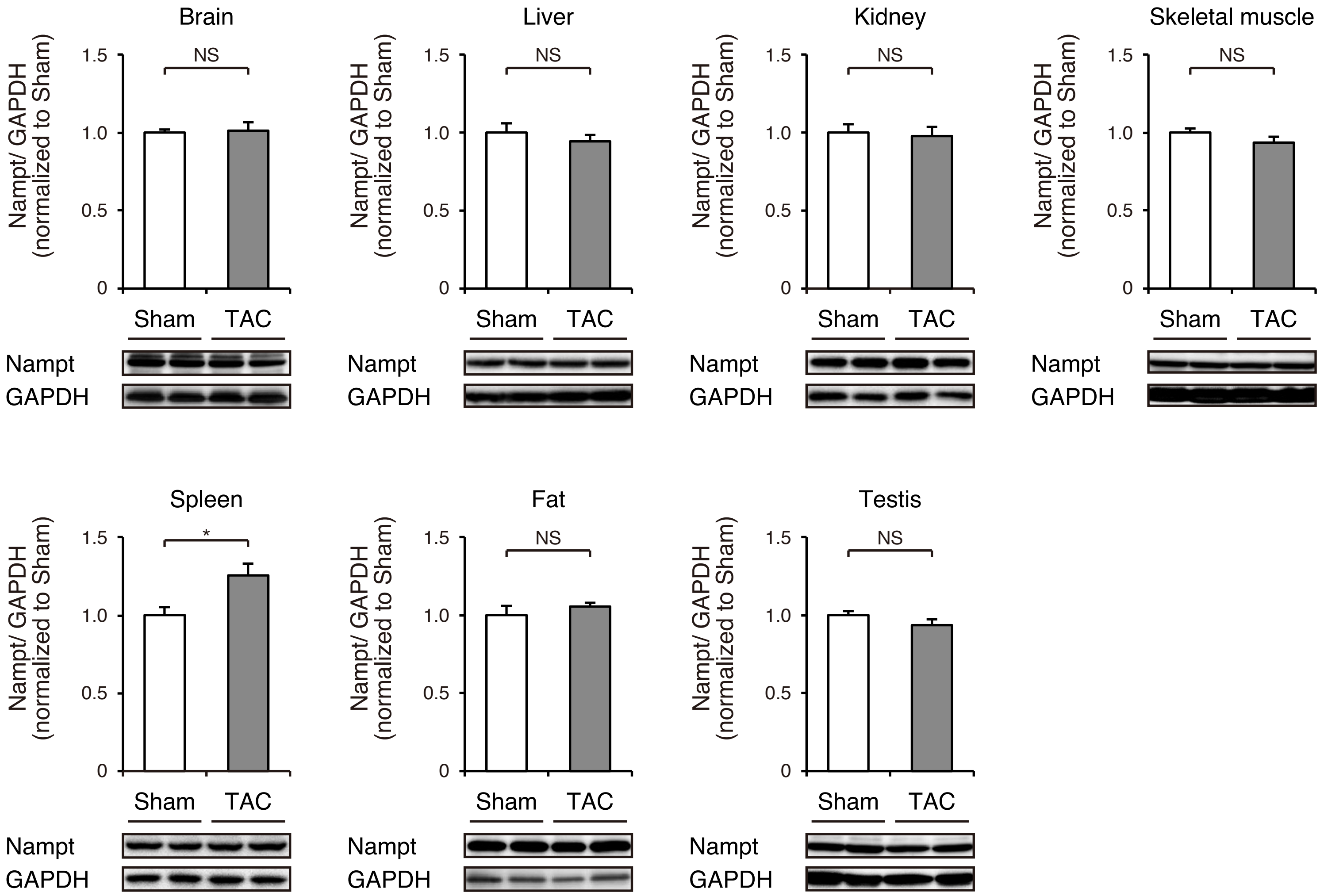

Supplementary Figure 1. Tissue distribution of Nampt in mice after TAC or sham operation
Immunoblot analysis of Nampt in mouse tissues at 8 w after TAC or sham operation (n = 4, in each group). Data are shown as fold induction over sham (mean $\pm$ SEM). *$P < 0.05$, NS, not significant.

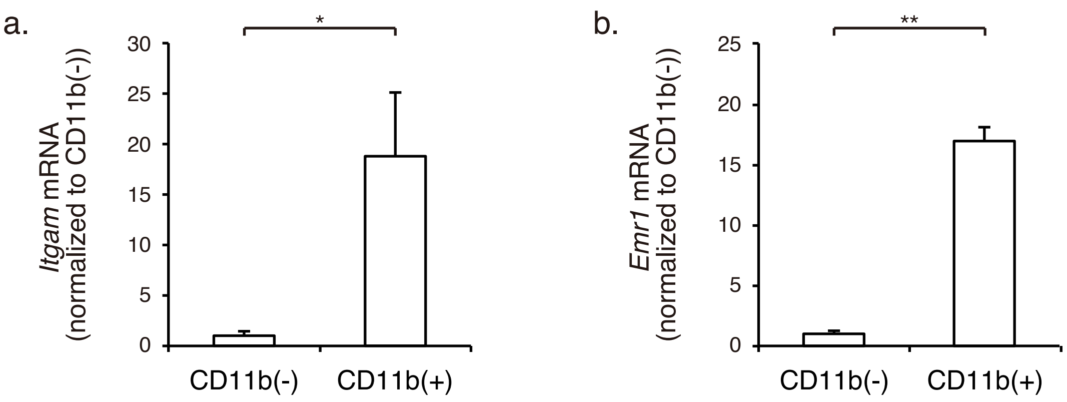

Supplementary Figure 2. Isolation of CD11b + mononuclear cells from peripheral blood of mice
The mRNA levels of *Itgam* (a) and *Emr1* (b) in CD11b + and CD11b – cells were quantified by real time RT-PCR analysis ($n = 4$, in each group). CD11b + cells were enriched by Histopaque-1083 (Sigma-Aldrich) centrifugation, followed by MACS (Miltenyi Biotec K.K.) positive selection of CD11b + using anti-rat CD11b antibody (Merck Millipore). Data are shown as fold induction over sham (mean ± SEM). $*P < 0.05$, $**P < 0.01$.

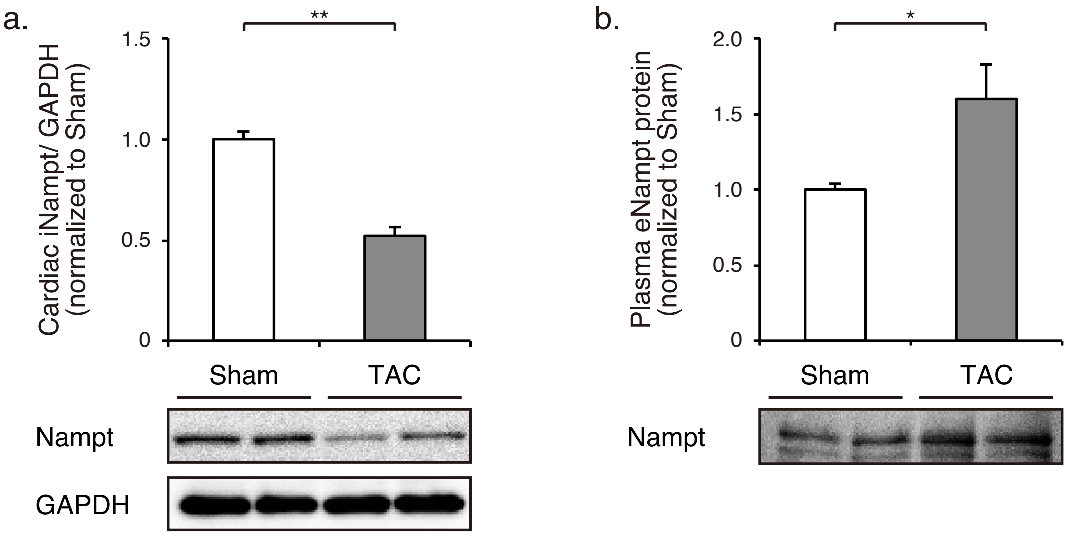

Supplementary Figure 3. Cardiac iNampt expression and plasma eNampt expression in mice at 1 w after TAC or sham operation

a. Immunoblot analysis of iNampt in the hearts of mice at 1 w after TAC or sham operation (n = 5, in each group). The quantitations of the Nampt/GAPDH are shown as bar graphs. Data are shown as fold induction over sham (mean $\pm$ SEM). $^{**}P < 0.01$.

b. Immunoblot analysis of eNampt in plasma of mice at 1 w after TAC or sham operation (n = 4, in each group). The quantitations of the eNampt are shown as bar graphs. Data are shown as fold induction over sham (mean $\pm$ SEM). $^{*}P < 0.05$.

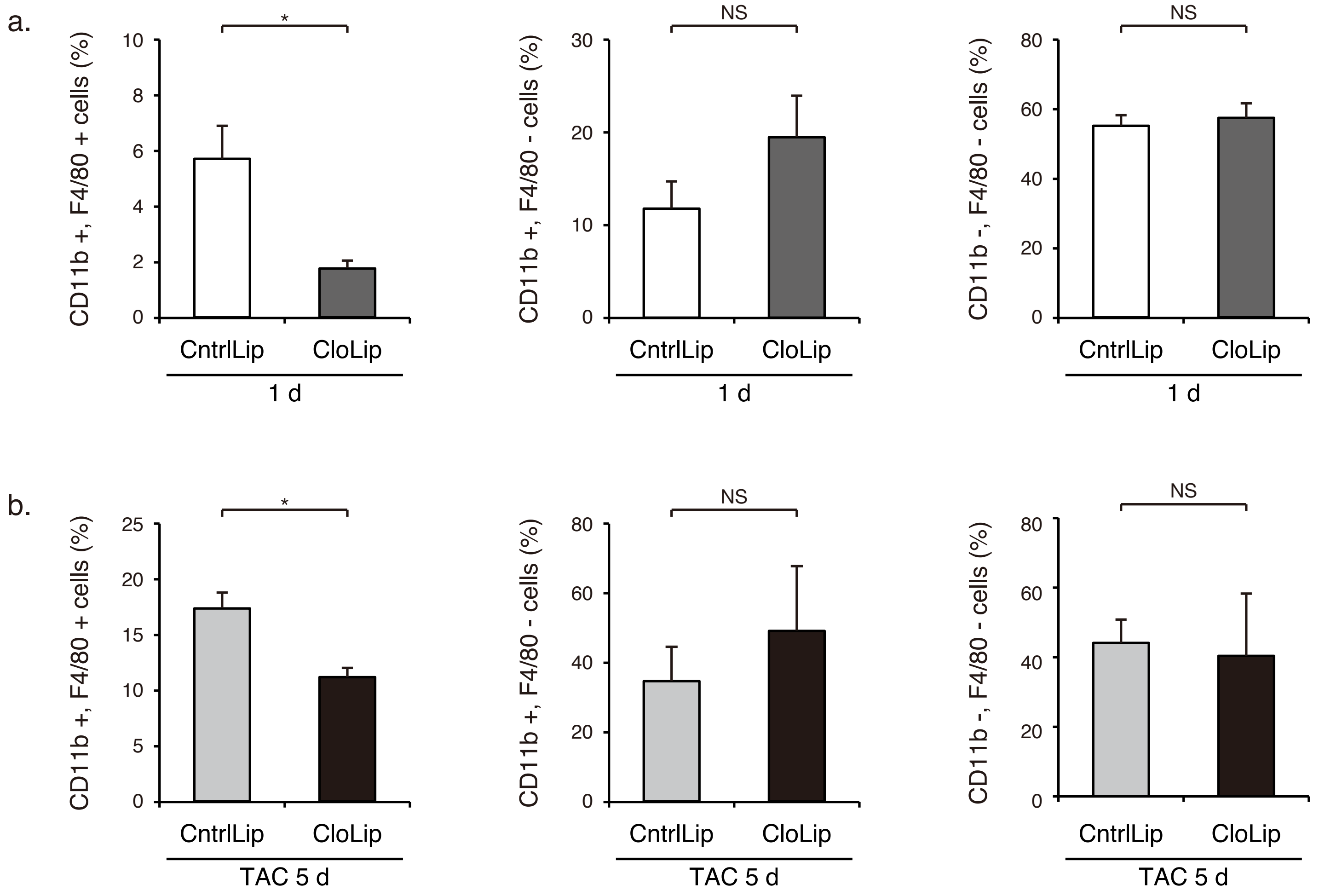

Supplementary Figure 4. The percentages of CD11b +, F4/80 + cells, CD11b +, F4/80 - cells, and CD11b -, F4/80 - cells in flow cytometric analysis of peripheral blood of mice

a. The percentages of CD11b +, F4/80 + cells, CD11b +, F4/80 - cells, and CD11b -, F4/80 - cells at 1 d after treatment with clodronate liposones (CloLip) (n = 3) or control liposomes (CntrlLip) (n = 3).
Data are shown mean ± SEM. *P < 0.05, NS, not significant.
b. The percentages of CD11b +, F4/80 + cells, CD11b +, F4/80 - cells, and CD11b -, F4/80 - cells at 5 d after TAC operation and treatment with CloLip (n = 3) or CntrlLip (n = 3). Data are shown mean ± SEM.
*P < 0.05, NS, not significant.

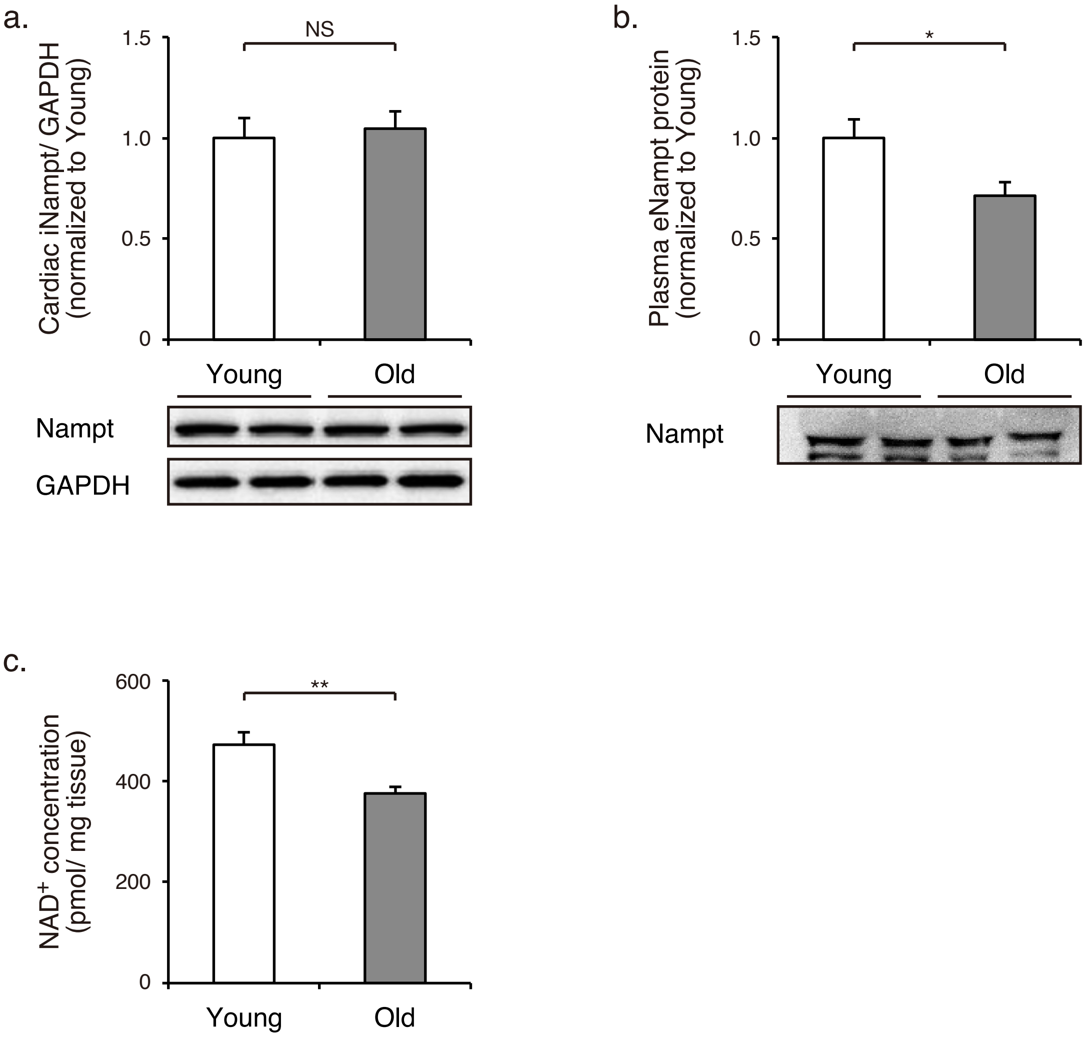

Supplementary Figure 5. Nampt-dependent biogensis of myocardial NAD$^+$ in young (8 w) and old (80-95 w) mice

a. Immunoblot analysis of iNampt in the hearts of mice young and old mice. The quantitations of the Nampt/GAPDH are shown as bar graphs. Data are shown as fold induction over young mice (mean $\pm$ SEM). NS, not significant.

b. Immunoblot analysis of eNampt in plasma of young and old mice. The quantitations of the eNampt are shown as bar graphs (n = 4, in each group). Data are shown as fold induction over young mice (mean $\pm$ SEM). *$P < 0.05$.

c. NAD$^+$ concentrations measured by HPLC in the hearts of young and old mice (n = 3, in each group). Data are presented as mean $\pm$ SEM. **$P < 0.01$.
